# Towards broadband artificial vision: CMOS-integrated SWIR-MWIR imaging

**DOI:** 10.1038/s41377-025-02087-3

**Published:** 2026-01-02

**Authors:** Di Sun, Wenxin Zheng, Hui Deng, Liangliang Liang

**Affiliations:** Institute of Flexible Electronics (IFE, Future Technologies), Xiang’an Campus, Xiamen University, Xiang’an South Road, 361102 Xiamen, Fujian China

**Keywords:** Optics and photonics, Physics

## Abstract

Inspired by the snake pit organ’s remarkable ability to perceive mid-wave infrared (MWIR) radiation, researchers have developed a biomimetic artificial vision system that integrates infrared-to-visible upconverters with CMOS sensors. Operating at room temperature, this platform enables direct visualization of both short-wave infrared (SWIR) and MWIR, marking a pioneering demonstration of broadband infrared imaging with high resolution. Such a breakthrough paves the way for low-cost and flexible applications in night vision, agricultural monitoring, industrial inspection, and beyond.

Artificial vision systems, drawing from nature’s diverse visual adaptations, have advanced rapidly to overcome limitations of conventional cameras^1^. These systems emulate biological traits such as the wide fields of view, superior object detection, foveated multispectral imaging, and panoramic vision, inspired by the eyes of aquatic animals^2^, felines^3^, birds^4^, and fiddler crabs^5^. However, most remain confined to the visible spectrum (0.4–0.78 μm), restricting their use in low-light, foggy, or dark environments.

Snakes offer a compelling model: their pit organs detect mid-wave infrared (MWIR, 3–5 μm) radiation, generating thermal images of prey even in complete darkness^6^ (Fig. 1a). In 2020, solution-processed colloidal quantum dot (CQD)-based upconverters achieved 6.5% efficiency, extending detection to near-infrared (NIR) and short-wave infrared (SWIR) up to 1.6 μm^7^. However, challenges persist: longer wavelengths increase dark current, degrading the signal-to-noise ratios at room temperature^8,9^. Additionally, inhomogeneous doping in CQD homojunctions hinders large-area fabrication, which is essential for high-resolution complementary metal-oxide semiconductor (CMOS) integration^10^.

**Fig. 1:**
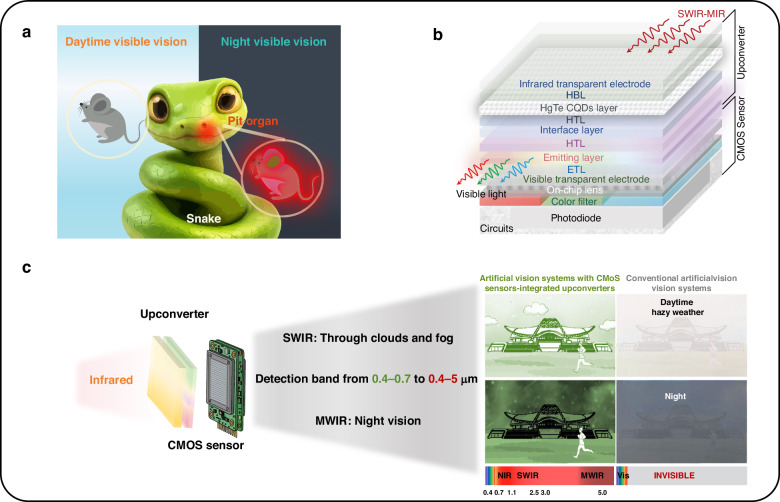
Working principle for CMOS-integrated SWIR-MWIR imaging. **a** Snake-inspired vision: snakes rely on their eyes in daylight and specialized pit organs to visualize mid-wave infrared radiation at night. **b** Artificial counterpart: schematic architecture of CMOS sensors monolithically integrated with quantum-dot upconverters for infrared-to-visible conversion. **c** Broadened capability: comparison of imaging outcomes, showing how CMOS-integrated artificial vision systems extend perception beyond the visible spectrum, unlike conventional cameras

In a recent study published in *Light: Science & Applications*, Mu and colleagues from Beijing Institute of Technology report a decisive advance. They designed HgTe CQD barrier heterojunction infrared detecting units and monolithically integrated them with CMOS sensors, achieving a broadband response spanning 1.1–5.0 μm at room temperature (Fig. 1b)^11^. Crucially, the ZnO/HgTe/P3HT interfacial barriers efficiently block the majority carrier dark current without sacrificing photocarrier transport, enabling zero-bias SWIR/MWIR detection with high signal-to-noise ratios. The system achieves upconversion efficiencies of 6.41% for SWIR (cut-off at 2.5 μm) and 4.06% for MWIR (cut-off at 4.5 μm).

A critical achievement is the wafer-level monolithic integration: organic light-emitting diodes and CQD detecting stacks were fabricated directly on commercial CMOS arrays via vacuum deposition and solution processing. The resulting device delivers 4K-resolution (3840 × 2160) imaging and 120 frames per second video-rate dynamic MWIR visualization, while maintaining performance under mechanical bending, showcasing its potential for flexible electronics. Demonstrations include SWIR imaging through silicon wafers and real-time MWIR thermal vision, underscoring both its sensitivity and practicality. Collectively, the detection band is extended from the visible (0.4–0.7 μm) to 0.4–4.5 μm, a roughly 14-fold expansion in spectral coverage (Fig. 1c).

This work sets a new benchmark for CMOS-compatible artificial vision by integrating mature silicon readouts with broadband infrared upconversion. Beyond cost-effective night vision, the combination of SWIR and MWIR channels with visible imaging could benefit autonomous driving^12^, food safety inspection^13^, environmental monitoring^14^, and aspects of medical diagnostics^15^. Looking ahead, key goals include further suppressing thermal noise toward longer wavelengths, boosting upconversion efficiency, and extending the response into the long-wave infrared regime. Progress will likely stem from the co-optimization of CQD chemistry, barrier-engineered device physics, and optical/electronic integration. Just as the snake’s pit organs enable it to navigate in total darkness, we foresee that similar artificial infrared vision technologies will soon empower a new generation of ubiquitous, flexible, and affordable imaging solutions.
